# Anti-acid therapy in idiopathic pulmonary fibrosis: insights from the INPULSIS® trials

**DOI:** 10.1186/s12931-018-0866-0

**Published:** 2018-09-03

**Authors:** Ulrich Costabel, Jürgen Behr, Bruno Crestani, Wibke Stansen, Rozsa Schlenker-Herceg, Susanne Stowasser, Ganesh Raghu

**Affiliations:** 1 Ruhrlandklinik, University Hospital, University of Duisburg-Essen, Essen, Germany; 2 Medizinische Klinik und Poliklinik V, University of Munich (LMU) and Asklepios Klinik München-Gauting, Member of the German Center for Lung Research, Munich, Germany; 3 APHP, Hôpital Bichat, Service de Pneumologie A, DHU FIRE; INSERM, Unité 1152; Université Paris Diderot, Paris, France; 4 Boehringer Ingelheim International GmbH, Ingelheim am Rhein, Germany; 5 Boehringer Ingelheim Pharmaceuticals Inc, Ridgefield, CT USA; 6 University of Washington, Seattle, WA USA

## Abstract

**Background:**

The benefits and risks of anti-acid medication in patients with idiopathic pulmonary fibrosis (IPF) remain a topic of debate. We investigated whether use of anti-acid medication at baseline was associated with differences in the natural course of disease or influenced the treatment effect of nintedanib in patients with IPF.

**Methods:**

*Post-hoc* analyses of outcomes in patients receiving versus not receiving anti-acid medication (proton pump or histamine-2 receptor inhibitor) at baseline using pooled data from the two Phase III randomized placebo-controlled INPULSIS® trials of nintedanib in patients with IPF.

**Results:**

At baseline, 406 patients were receiving anti-acid medication (244 nintedanib; 162 placebo) and 655 were not (394 nintedanib; 261 placebo). In an analysis of the natural course of IPF by anti-acid medication use at baseline, the adjusted annual rate of decline in FVC was − 252.9 mL/year in placebo-treated patients who were receiving anti-acid medication at baseline and − 205.4 mL/year in placebo-treated patients who were not (difference of − 47.5 mL/year [95% CI: –105.1, 10.1]; *p* = 0.1057). In an analysis of the potential influence of anti-acid medication use on the treatment effect of nintedanib, the adjusted annual rates of decline in FVC were − 124.4 mL/year in the nintedanib group and − 252.9 mL/year in the placebo group (difference of 128.6 mL/year [95% CI: 74.9, 182.2]) in patients who were receiving anti-acid medication at baseline and − 107.0 mL/year in the nintedanib group and − 205.3 mL/year in the placebo group (difference of 98.3 mL/year [95% CI: 54.1, 142.5]) in patients who were not (treatment-by-time-by-subgroup interaction *p* = 0.3869). The proportions of patients who had ≥1 investigator-reported acute exacerbation were 11.7% and 5.0% in placebo-treated patients, and 4.9% and 4.8% of nintedanib-treated patients, among patients who were and were not receiving anti-acid medication at baseline, respectively.

**Conclusions:**

In *post-hoc* analyses of data from the INPULSIS® trials, anti-acid medication use at baseline was not associated with a more favorable course of disease, and did not impact the treatment effect of nintedanib, in patients with IPF.

**Trial registration:**

ClinicalTrials.gov identifiers: NCT01335464 and NCT01335477.

**Electronic supplementary material:**

The online version of this article (10.1186/s12931-018-0866-0) contains supplementary material, which is available to authorized users.

## Background

Idiopathic pulmonary fibrosis (IPF) is a specific form of progressive fibrosing interstitial lung disease characterized by worsening lung function and dyspnea [1]. IPF is believed to develop as a result of an aberrant wound healing response to epithelial injury, in which activated alveolar epithelial cells release fibrogenic growth factors, promoting fibroblast proliferation and transformation to myofibroblasts and excess deposition of extracellular matrix [2]. IPF typically presents during the sixth or seventh decade and is more common in men than in women [1]. The natural history of IPF is variable but its prognosis is poor [3]. Acute deteriorations in respiratory function, known as acute exacerbations, are a common feature of the clinical course of IPF and a major cause of morbidity and mortality [4].

Gastroesophageal reflux disease (GERD) is known to be a common comorbidity in patients with IPF, but its reported prevalence varies widely according to the definition used [5]. In the INSIGHTS-IPF registry of patients with IPF in Germany, 29% of patients reported GERD on enrollment [6], while in a prospective study of 65 patients with IPF, acid gastroesophageal reflux occurred in 87% of patients subjected to 24-h pH monitoring [7]. It has been hypothesized that microaspiration caused by GERD plays a role in the pathogenesis of IPF by causing injury to the lung epithelium and the initiation of inflammatory cascades [8–11] but a causal relationship between GERD and IPF has not been established.

Proton pump inhibitors used to treat GERD have pleiotropic antioxidant and anti-inflammatory effects [12]. Proton pump inhibitors target the H+/K+ ATPase, which has recently been shown to be expressed in the hyperplastic alveolar epithelium in patients with IPF [13]. Use of anti-acid medication has been associated with a decreased rate of decline in FVC and improved survival in some observational studies in patients with IPF [14, 15]. Surgical intervention to treat GERD (i.e. Nissen fundoplication) in patients with IPF has also been associated with improved survival [14, 16]. The latest international clinical practice guidelines for IPF included a conditional recommendation for the use of anti-acid therapy in patients with asymptomatic GERD, based on very low quality evidence [17]. However, there are no data from randomized controlled trials showing an improvement in outcomes in patients with IPF treated with anti-acid medication and the benefits and risks of anti-acid medication in patients with IPF remain a topic of debate [18–20].

Nintedanib is an intracellular inhibitor of tyrosine kinases that has been approved for the treatment of IPF in many countries and regions, including the US, Europe and Asia. In the latest international clinical practice guidelines for IPF [17], nintedanib received a conditional recommendation for use based on data from three randomized controlled trials [21, 22]. In the two replicate Phase III INPULSIS® trials, nintedanib slowed disease progression in patients with IPF by reducing the rate of decline in forced vital capacity (FVC) by about 50% [22]. The most frequently reported adverse events in patients treated with nintedanib were gastrointestinal, particularly diarrhea.

We conducted *post-hoc* analyses of data from the INPULSIS® trials to investigate whether use of anti-acid medication at baseline was associated with differences in the natural course of disease or influenced the treatment effect of nintedanib in patients with IPF.

## Methods

The design of the INPULSIS® trials has been described [22]. Briefly, participants had to have been diagnosed with IPF within the previous 5 years and to have an FVC of ≥50% predicted and a diffusing capacity of the lungs for carbon monoxide (DLco) of 30–79% predicted. In the absence of a surgical lung biopsy, patients were required to have honeycombing and/or a combination of traction bronchiectasis and reticulation in the absence of atypical features of usual interstitial pneumonia on high-resolution computed tomography (HRCT). Patients were randomized 3:2 to receive nintedanib 150 mg bid or placebo for 52 weeks, with a follow-up visit 4 weeks later. The primary endpoint was the annual rate of decline in FVC (mL/year), analyzed using a random coefficient regression model including sex, age and height as covariates. Key secondary endpoints were time to first investigator-reported acute exacerbation (defined in [22]) and change from baseline in St George’s Respiratory Questionnaire (SGRQ) total score, both over 52 weeks. Time to first acute exacerbation was analyzed using the log rank test, with hazard ratios and confidence intervals obtained using the Cox’s proportional hazards model adjusted for sex, age and height. Change from baseline in SGRQ total score was analyzed using a mixed model for repeated measures including treatment and visit as fixed effects, baseline score as a covariate, and treatment-by-visit and baseline score-by-visit as interaction terms.

The *post-hoc* analyses presented in this paper were based on pooled data from both INPULSIS® trials and included all patients who received ≥1 dose of study drug. Anti-acid medications comprised proton pump inhibitors and histamine-2 receptor antagonists. Patients who took anti-acid medication before and also after the first intake of trial medication (based on case report forms) were considered to be using anti-acid medication at baseline. The presence of GERD was assessed solely based on the comorbidities reported by the patient and provided in the case report form.

To evaluate whether anti-acid medication use at baseline was associated with differences in the natural course of IPF, analyses of the annual rate of decline in FVC (mL/year) and the time to absolute decline in FVC ≥10% predicted or death over 52 weeks were conducted in patients treated with placebo who were versus were not receiving anti-acid medication at baseline. A Cox regression model with terms for trial, subgroup, sex, age and height was used to assess time to absolute decline in FVC ≥10% predicted or death over 52 weeks. The original statistical approach for the annual rate of decline in FVC was repeated, but with an interaction term time-by-baseline anti-acid medication use included as a fixed effect in the model.

To evaluate the potential influence of anti-acid medication at baseline on the treatment effect of nintedanib, analyses of the annual rate of decline in FVC (mL/year), time to first investigator-reported acute exacerbation, change from baseline in SGRQ total score, time to absolute decline in FVC ≥5% predicted or death, or FVC ≥10% predicted or death, over 52 weeks were conducted in patients who were versus were not receiving anti-acid medication at baseline. For the annual rate of decline in FVC, the term subgroup and the interaction terms treatment-by-subgroup, time-by-subgroup and treatment-by-time-by-subgroup were included in the original model. For time to first investigator-reported acute exacerbation and change from baseline in SGRQ total score, the term subgroup and the interaction term treatment-by-subgroup were included in the model. Time to absolute decline in FVC ≥5% predicted or death, or FVC ≥10% predicted or death was analyzed using a Cox regression model with the term subgroup and the interaction term treatment-by-subgroup included in the model. The interaction *p*-value is an indicator of the potential difference in the treatment effect of nintedanib versus placebo between the subgroups. Statistical Analysis System (SAS) version 9.4 was used for all the analyses.

Safety was assessed via clinical and laboratory evaluation and the recording of adverse events with onset after the first dose and up to 28 days after the last dose of study drug. Adverse events were coded according to the Medical Dictionary for Regulatory Activities version 16.1. Safety analyses were descriptive.

## Results

### Patients

A total of 406 (38%) patients were receiving anti-acid medication at baseline (244 in the nintedanib group, 162 in the placebo group) and 655 patients were not (394 in the nintedanib group, 261 in the placebo group). Of the patients receiving anti-acid medication at baseline, 226 (93%) in the nintedanib group and 147 (91%) in the placebo group were receiving ≥1 proton pump inhibitor; 25 patients (10%) in the nintedanib group and 22 patients (14%) in the placebo group were receiving ≥1 histamine-2 receptor antagonist (Additional file 1).

Baseline characteristics were generally similar between the subgroups by use of anti-acid medication use at baseline, but a greater proportion of White than Asian patients were receiving anti-acid medication and SGRQ total score was higher (indicating worse quality of life) in patients receiving anti-acid medication (Table 1). Of the patients receiving anti-acid medication at baseline, 52.0% and 51.2% of patients in the nintedanib and placebo groups, respectively, reported having GERD (Additional file 2).

**Table 1:** Baseline characteristics

	Anti-acid medication at baseline		No anti-acid medication at baseline	
	Nintedanib (*n* = 244)	Placebo (*n* = 162)	Nintedanib (*n* = 394)	Placebo (*n* = 261)
Age, years, mean (SD)	67.4 (8.3)	68.0 (7.4)	66.1 (8.0)	66.4 (8.1)
Male, n (%)	179 (73.4)	124 (76.5)	328 (83.2)	210 (80.5)
Race, n (%)				
White	164 (67.2)	117 (72.2)	196 (49.7)	131 (50.2)
Asian	40 (16.4)	28 (17.3)	154 (39.1)	100 (38.3)
Black	0 (0.0)	0 (0.0)	2 (0.5)	0 (0.0)
Missing*	40 (16.4)	17 (10.5)	42 (10.7)	30 (11.5)
Former or current smoker, n (%)	170 (69.7)	121 (74.7)	294 (74.6)	180 (69.0)
FVC, mL, mean (SD)	2643 (712)	2679 (818)	2757 (781)	2758 (806)
FVC, % predicted, mean (SD)	80.3 (17.4)	78.4 (17.3)	79.4 (17.7)	79.8 (18.8)
FEV_1_/FVC, %, mean (SD)	82.0 (5.5)	81.9 (5.9)	81.4 (6.1)	81.5 (6.0)
DLco, % predicted, mean (SD)	47.6 (15.1)	48.1 (14.3)	47.3 (12.4)	46.3 (12.8)
SGRQ total score, mean (SD)^†^	43.4 (18.7)	44.1 (17.9)	37.2 (19.1)	36.8 (18.4)

*In France, regulation did not permit the collection of data on race. ^†^*n* = 234 for nintedanib and *n* = 160 for placebo in anti-acid medication at baseline subgroup; *n* = 390 for nintedanib and *n* = 259 for placebo in no anti-acid medication at baseline subgroup

In patients receiving anti-acid medication at baseline, mean (SD) duration of exposure to study medication was 10.1 (3.5) months in the nintedanib group and 10.7 (2.8) months in the placebo group. In patients not receiving anti-acid medication at baseline, mean (SD) duration of exposure in these groups was 10.4 (3.3) months and 10.9 (2.8) months, respectively.

### Annual rate of decline in FVC

In the evaluation of whether there was a difference in the natural course of IPF by anti-acid medication use at baseline, the adjusted annual rate of decline in FVC was − 252.9 mL/year in placebo-treated patients who were receiving anti-acid medication at baseline and − 205.4 mL/year in placebo-treated patients who were not receiving anti-acid medication at baseline (difference of − 47.5 mL/year [95% CI: –105.1, 10.1]; *p* = 0.1057).

**Fig. 1:**
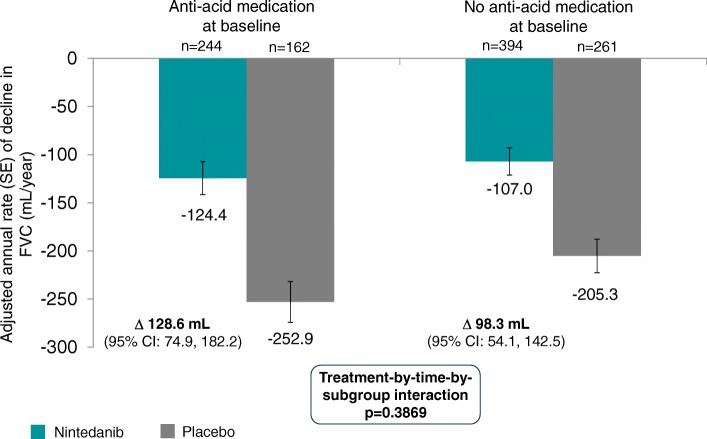
Annual rate of decline in FVC

In analyses to investigate the potential influence of anti-acid medication use at baseline on the treatment effect of nintedanib, the adjusted annual rates of decline in FVC were − 124.4 mL/year in the nintedanib group and − 252.9 mL/year in the placebo group (difference of 128.6 mL/year [95% CI: 74.9, 182.2]) in patients who were receiving anti-acid medication at baseline and − 107.0 mL/year in the nintedanib group and − 205.3 mL/year in the placebo group (difference of 98.3 mL/year [95% CI: 54.1, 142.5]) in patients who were not (Fig. 1). There was no significant treatment-by-time-by-subgroup interaction (*p* = 0.3869), indicating that the treatment effect was the same in both subgroups (Fig. 1).

### Disease progression

There was a numerical but not significant difference in the time to absolute decline in FVC ≥10% predicted or death between placebo-treated patients who were taking anti-acid medication at baseline versus were not receiving anti-acid medication at baseline (HR 1.33 [95% CI: 0.98, 1.80]; *p* = 0.0661). In total, 47.5% and 37.5% of placebo-treated patients who were and were not receiving anti-acid medication at baseline, respectively, had a decline in FVC ≥10% predicted or died, while 75.3% and 69.3% of placebo-treated patients in these subgroups, respectively, had a decline in FVC ≥5% predicted or died.

In analyses to investigate the potential influence of anti-acid medication use at baseline on the treatment effect of nintedanib, the HRs for time to decline in FVC ≥10% predicted or death were 0.60 (95% CI: 0.43, 0.82) in patients who were receiving anti-acid medication at baseline and 0.60 (95% CI: 0.45, 0.79) in patients who were not (treatment-by-subgroup interaction *p* = 0.9808). The HRs for time to decline in FVC ≥5% predicted or death were 0.61 (95% CI: 0.48, 0.78) and 0.60 (95% CI: 0.49, 0.73) in these groups, respectively (treatment-by-subgroup interaction *p* = 0.8661). For both disease progression endpoints, the criterion reached was FVC decline rather than death in most patients (Table 2).

**Table 2:** Disease progression

N (%)	Anti-acid medication at baseline		No anti-acid medication at baseline	
	Nintedanib (n = 244)	Placebo (n = 162)	Nintedanib (n = 394)	Placebo (n = 261)
Absolute decline in FVC ≥10% predicted or death	77 (31.6)	77 (47.5)	96 (24.4)	98 (37.5)
Criterion reached first				
Absolute decline in FVC ≥10% predicted	67 (27.5)	65 (40.1)	81 (20.6)	88 (33.7)
Death	10 (4.1)	12 (7.4)	15 (3.8)	10 (3.8)
Absolute decline in FVC ≥5% predicted or death	135 (55.3)	122 (75.3)	195 (49.5)	181 (69.3)
Criterion reached first				
Absolute decline in FVC ≥5% predicted	129 (52.9)	117 (72.2)	188 (47.7)	175 (67.0)
Death	6 (2.5)	5 (3.1)	7 (1.8)	6 (2.3)

### Investigator-reported acute exacerbations

**Fig. 2:**
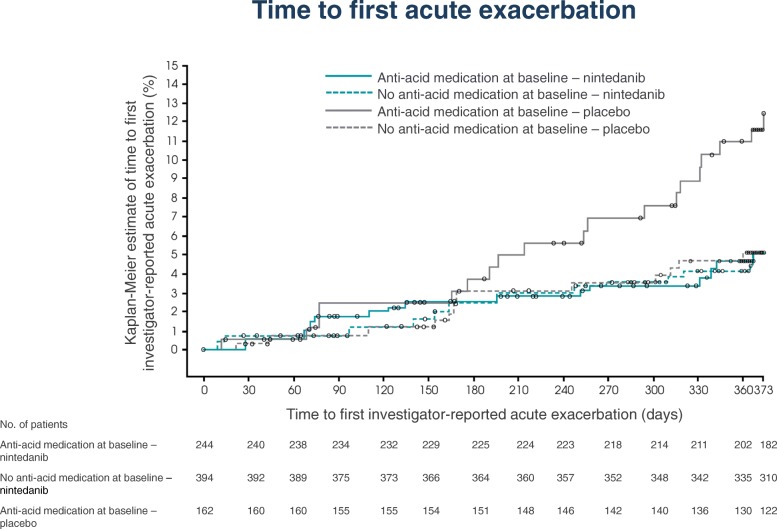
Time to first investigator-reported acute exacerbation

Kaplan-Meier estimates of time to first acute exacerbation are presented in Fig. 2. In the placebo group, 19 patients (11.7%) and 13 patients (5.0%) who were and were not receiving anti-acid medication at baseline, respectively, had ≥1 acute exacerbation. In the nintedanib group, 12 patients (4.9%) and 19 patients (4.8%) who were and were not receiving anti-acid medication at baseline, respectively, had ≥1 acute exacerbation. The HR for nintedanib versus placebo for time to first acute exacerbation was 0.40 (95% CI: 0.19, 0.83) in patients who were receiving anti-acid medication at baseline and 0.99 (95% CI: 0.49, 2.00) in patients who were not. Although there was no significant treatment-by-subgroup interaction (*p* = 0.0949), there appeared to be a numerical difference, not ruling out a potential difference in the treatment effect of nintedanib between the subgroups.

### SGRQ total score

**Fig. 3:**
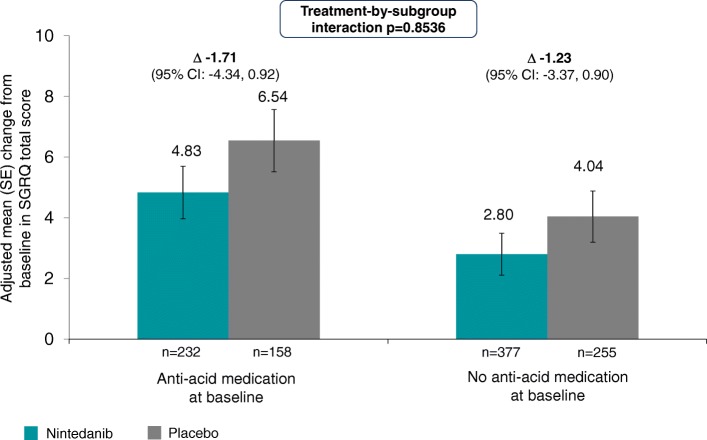
Change from baseline in SGRQ total score

In the placebo group, the adjusted mean changes from baseline in SGRQ total score over 52 weeks were 6.54 and 4.04 in patients who were and were not receiving anti-acid medication at baseline, respectively; in the nintedanib group, the adjusted mean changes from baseline were 4.83 and 2.80 in these subgroups, respectively (Fig. 3). There was no significant treatment-by-subgroup interaction (*p* = 0.8536), indicating that the treatment effect was the same in both subgroups.

### Adverse events

A summary of adverse events is presented in Table 3. Diarrhea was the most frequent adverse event in nintedanib-treated patients, reported in 60.7% and 63.5% of patients who were and were not receiving anti-acid medication at baseline compared with 17.3% and 19.2% of placebo-treated patients in these groups, respectively. Permanent discontinuation of study medication due to diarrhea was more frequent in nintedanib-treated patients who were receiving anti-acid medication at baseline than those were not (6.6% versus 3.0%). Pneumonia was reported in 5.3% and 7.4% of nintedanib- and placebo-treated patients who were receiving anti-acid medication at baseline compared with 4.1% and 4.6% of nintedanib- and placebo-treated patients who were not, respectively. Serious adverse events were reported in 35.7% and 37.0% of nintedanib- and placebo-treated patients who were receiving anti-acid medication at baseline compared with 27.2% and 25.7% of nintedanib- and placebo-treated patients who were not, respectively (Additional file 3).

**Table 3:** Adverse events

	Anti-acid medication at baseline		No anti-acid medication at baseline	
	Nintedanib (n = 244)	Placebo (n = 162)	Nintedanib (n = 394)	Placebo (n = 261)
Any adverse event(s)	235 (96.3)	152 (93.8)	374 (94.9)	227 (87.0)
Most frequent adverse event(s)*				
Diarrhea	148 (60.7)	28 (17.3)	250 (63.5)	50 (19.2)
Nausea	66 (27.0)	14 (8.6)	90 (22.8)	14 (5.4)
Nasopharyngitis	32 (13.1)	24 (14.8)	55 (14.0)	44 (16.9)
Cough	41 (16.8)	29 (17.9)	44 (11.2)	28 (10.7)
Vomiting	36 (14.8)	4 (2.5)	38 (9.6)	7 (2.7)
Decreased appetite	27 (11.1)	9 (5.6)	41 (10.4)	15 (5.7)
Bronchitis	35 (14.3)	18 (11.1)	32 (8.1)	27 (10.3)
Progression of IPF^†^	28 (11.5)	30 (18.5)	36 (9.1)	31 (11.9)
Weight decreased	26 (10.7)	5 (3.1)	36 (9.1)	10 (3.8)
Upper respiratory tract infection	20 (8.2)	18 (11.1)	38 (9.6)	24 (9.2)
Dyspnea	23 (9.4)	22 (13.6)	26 (6.6)	26 (10.0)
Headache	25 (10.2)	8 (4.9)	18 (4.6)	11 (4.2)
Serious adverse event(s)^‡^	87 (35.7)	60 (37.0)	107 (27.2)	67 (25.7)
Severe adverse event(s)^§^	84 (34.4)	44 (27.2)	90 (22.8)	55 (21.1)
Fatal adverse event(s)	20 (8.2)	15 (9.3)	17 (4.3)	16 (6.1)
Adverse event(s) leading to treatment discontinuation^¶^	57 (23.4)	26 (16.0)	66 (16.8)	29 (11.1)
Diarrhea	16 (6.6)	0 (0.0)	12 (3.0)	1 (0.4)
Progression of IPF^†^	5 (2.0)	12 (7.4)	8 (2.0)	9 (3.4)
Nausea	4 (1.6)	0 (0.0)	9 (2.3)	0 (0.0)
Pneumonia	5 (2.0)	1 (0.6)	1 (0.3)	0 (0.0)

Data shown are n(%) of patients in whom ≥1 such event was reported

*Adverse events reported in ≥10% of patients in any of the subgroups shown

^†^Corresponds to Medical Dictionary for Regulatory Activities (MedDRA) term ‘IPF’, which included disease worsening and acute exacerbations of IPF

^‡^An event that resulted in death, was immediately life-threatening, resulted in persistent or clinically significant disability or incapacity, required or prolonged hospitalization, was related to a congenital anomaly or birth defect, or was deemed serious for any other reason

^§^An event that was incapacitating or that caused an inability to work or to perform usual activities

^¶^Adverse events leading to treatment discontinuation in ≥2% of patients in any of the subgroups shown

## Discussion

The results of this *post-hoc* subgroup analysis of data from the INPULSIS® trials showed that use of anti-acid medication at baseline generally did not influence the treatment effect of nintedanib in patients with IPF. In the placebo group, the annual rate of decline in FVC was numerically higher in patients who were receiving anti-acid medication at baseline than in those who were not (− 252.9 vs − 205.4 mL/year; difference of − 47.5 mL/year [95% CI: –105.1, 10.1]; *p* = 0.1057) and a greater proportion of patients receiving anti-acid medication at baseline experienced decline in FVC ≥10% predicted or died over 52 weeks (47.5% vs 37.5%). These findings should be interpreted with caution given that these analyses were conducted *post-hoc*; that patients were not randomized according to reported use of anti-acid medication; that information on the type, dose, and duration of anti-acid medication used throughout (or prior to) the study are lacking; that the efficacy of anti-acid medication in suppressing acid reflux is not known, and that it is unknown whether the patients were taking anti-acid medication to treat symptomatic GERD (acid or non-acid), assumed silent GERD, or even IPF. Similar caveats apply to the interpretation of data from a *post-hoc* analysis of pooled data from three randomized trials of pirfenidone in patients with IPF, in which use of anti-acid therapy at baseline was not associated with a difference in the occurrence of disease progression in either the placebo group [18, 23] or the pirfenidone group [24].

Elevated pepsin levels in bronchoalveolar lavage fluid have been documented in patients manifesting acute exacerbations of IPF [8]. In a prospective observational study of data from placebo-treated patients with IPF in the IPFnet trials, there were no adjudicated acute exacerbations in the 124 patients who used anti-acid medication at baseline (of whom 117 continued to take it for the entire study period) versus 9 acute exacerbations in the 118 patients who did not use anti-acid medication at baseline [15]. In contrast, in our *post-hoc* analysis of data from the INPULSIS® trials, acute exacerbations reported by the site investigators occurred in a greater proportion of placebo-treated patients who were receiving anti-acid medication at baseline than in those who were not (11.7% [19 patients] versus 5.0% [13 patients]). An increased risk of investigator-reported acute exacerbations (and adjudicated confirmed or suspected acute exacerbations) was also observed in a risk factor analysis based on pooled data from these trials [25]. There are multiple hypotheses that may explain these findings. Patients who were receiving anti-acid medication at baseline may have had a greater extent of IPF than patients who were not. The patients receiving anti-acid medication at baseline had worse health-related quality of life at baseline according to SGRQ total score and a greater frequency of fatal adverse events, but did not have greater lung function impairment based on FVC or DLco. Secondly, it is possible that the increased frequency of acute exacerbations in patients taking anti-acid medication at baseline may be due to insufficiently controlled microaspiration in patients who had co-existing GERD. Abnormal acid gastro-oesphageal reflux has been documented in patients with IPF taking proton pump inhibitors [26], and the use of antiacid medication may not have fully suppressed acid reflux in patients in these trials who had concomitant GERD. Thirdly, it may be hypothesized that patients receiving anti-acid medication at baseline were at greater risk of infectious respiratory events (e.g. pneumonia) due to altered host defense against bacteria as a result of increased gastric pH. In our analysis, pneumonia was reported in a numerically higher proportion of placebo-treated patients receiving anti-acid medication at baseline than placebo-treated patients who were not. Epidemiological data suggest a potential adverse relationship between proton pump inhibitor use and community acquired pneumonia [27–29], and a *post-hoc* analysis of data from placebo-treated patients in trials of pirfenidone suggested that in patients with FVC < 70% predicted at baseline, unadjudicated pulmonary infections were more common in patients who were taking anti-acid medication at baseline [18, 19, 23].

The proportion of patients experiencing diarrhea adverse events was similar in the subgroups by anti-acid medication use at baseline, but in the nintedanib group, the frequency of permanent treatment discontinuations due to diarrhea, although low, was numerically higher in patients receiving anti-acid medication at baseline. Overall, the adverse event profile of nintedanib in patients in both subgroups by anti-acid medication use was as expected based on the safety and tolerability profile reported in the overall patient population [22].

## Conclusion

In conclusion, this *post-hoc* analysis of data from the INPULSIS® trials showed that anti-acid medication use at baseline was not associated with a more favorable course of disease, and did not impact the treatment effect of nintedanib, in patients with IPF. Several hypotheses generated from this data warrant additional research, including prospective randomized clinical trials, to determine the role of acid and non-acid GERD in patients with interstitial lung diseases such as IPF and to characterize the benefits and risks of anti-acid medication in these patients. Further, the results of the WRAP-IPF trial (NCT01982968) will provide insights regarding the effects of laparoscopic anti-reflux surgery in patients with IPF.

## Additional files

Additional file 1: Proton pump inhibitors and histamine-2 receptor antagonists used at baseline in patients taking anti-acid medications at baseline. (DOCX 17 kb)

Additional file 2: Comorbidities reported at baseline. (DOCX 18 kb)

Additional file 3: Severe, serious and fatal adverse events by subgroups. (DOCX 19 kb)

## Abbreviations

DLco: Diffusing capacity of the lungs for carbon monoxide
FVC: Forced vital capacity
GERD: Gastroesophageal reflux disease
HR: Hazard ratio
HRCT: High-resolution computed tomography
IPF: Idiopathic pulmonary fibrosis
SD: Standard deviation
SGRQ: St George’s Respiratory Questionnaire
