# Supplementary material for: Anti-acid therapy in idiopathic pulmonary fibrosis: insights from the INPULSIS® trials
Source: Respir Res. 2018 Sep 3;19:167. doi: 10.1186/s12931-018-0866-0 (PMC6122773; doi:10.1186/s12931-018-0866-0)
Supplement: Supplementary file 1 — Proton pump inhibitors and histamine-2 receptor antagonists used at baseline in patients taking anti-acid medications at baseline. (DOCX 17 kb) [file 12931_2018_866_MOESM1_ESM.docx]

**Additional file 1**

**Proton pump inhibitors and histamine-2 receptor antagonists used at baseline in patients taking anti-acid medications at baseline**

|  | Nintedanib  (n=244) | Placebo  (n=162) |
| --- | --- | --- |
| ≥1 proton pump inhibitor, n (%) | 226 (100.0) | 147 (100.0) |
| Omeprazole | 94 (41.6) | 57 (38.8) |
| Esomeprazole magnesium | 40 (17.7) | 20 (13.6) |
| Lansoprazole | 40 (17.7) | 27 (18.4) |
| Pantoprazole sodium sesquihydrate | 20 (8.8) | 24 (16.3) |
| Rabeprazole sodium | 13 (5.8) | 6 (4.1) |
| Pantoprazole | 9 (4.0) | 5 (3.4) |
| Esomeprazole | 7 (3.1) | 6 (4.1) |
| Rabeprazole | 3 (1.3) | 0 (0.0) |
| Esomeprazole strontium | 1 (0.4) | 0 (0.0) |
| Dexlansoprazole | 0 (0.0) | 1 (0.7) |
| Esomeprazole sodium | 0 (0.0) | 1 (0.7) |
| ≥1 histamine-2 receptor antagonist, n (%) | 25 (100.0) | 22 (100.0) |
| Famotidine | 10 (40.0) | 8 (36.4) |
| Ranitidine hydrochloride | 6 (24.0) | 8 (36.4) |
| Ranitidine | 5 (20.0) | 3 (13.6) |
| Lafutidine | 3 (12.0) | 1 (4.5) |
| Nizatidine | 1 (4.0) | 2 (9.1) |
| Cimetidine | 0 (0.0) | 1 (4.5) |
