# Supplementary material for: Anti-acid therapy in idiopathic pulmonary fibrosis: insights from the INPULSIS® trials
Source: Respir Res. 2018 Sep 3;19:167. doi: 10.1186/s12931-018-0866-0 (PMC6122773; doi:10.1186/s12931-018-0866-0)
Supplement: Supplementary file 2 — Comorbidities reported at baseline. (DOCX 18 kb) [file 12931_2018_866_MOESM2_ESM.docx]

**Additional file 2 Comorbidities reported at baseline**

|  | Anti-acid medication  at baseline | | No anti-acid medication  at baseline | |
| --- | --- | --- | --- | --- |
|  | Nintedanib (n=244) | Placebo (n=162) | Nintedanib (n=394) | Placebo (n=261) |
| Hypertension | 116 (47.5) | 83 (51.2) | 160 (40.6) | 91 (34.9) |
| GERD | 127 (52.0) | 83 (51.2) | 20 (5.1) | 18 (6.9) |
| Diabetes mellitus | 29 (11.9) | 21 (13.0) | 59 (15.0) | 28 (10.7) |
| Hypercholesterolemia | 49 (20.1) | 38 (23.5) | 38 (9.6) | 33 (12.6) |
| Hyperlipidemia | 28 (11.5) | 25 (15.4) | 58 (14.7) | 13 (5.0) |
| Osteoarthritis | 40 (16.4) | 23 (14.2) | 33 (8.4) | 29 (11.1) |
| Benign prostatic hyperplasia | 23 (9.4) | 21 (13.0) | 31 (7.9) | 24 (9.2) |
| Sleep apnea syndrome | 26 (10.7) | 23 (14.2) | 26 (6.6) | 16 (6.1) |
| Depression | 31 (12.7) | 26 (16.0) | 21 (5.3) | 14 (5.4) |
| Type 2 diabetes mellitus | 22 (9.0) | 17 (10.5) | 23 (5.8) | 21 (8.0) |
| Dyslipidemia | 26 (10.7) | 6 (3.7) | 19 (4.8) | 19 (7.3) |
| Coronary artery disease | 16 (6.6) | 21 (13.0) | 29 (7.4) | 22 (8.4) |
| Cataract | 12 (4.9) | 17 (10.5) | 23 (5.8) | 16 (6.1) |
| Hiatus hernia | 27 (11.1) | 17 (10.5) | 4 (1.0) | 0 (0.0) |

Conditions reported in ≥10% of patients in any of the subgroups are shown.
