# Supplementary material for: Anti-acid therapy in idiopathic pulmonary fibrosis: insights from the INPULSIS® trials
Source: Respir Res. 2018 Sep 3;19:167. doi: 10.1186/s12931-018-0866-0 (PMC6122773; doi:10.1186/s12931-018-0866-0)
Supplement: Supplementary file 3 — Severe, serious and fatal adverse events by subgroups. (DOCX 19 kb) [file 12931_2018_866_MOESM3_ESM.docx]

**Additional file 3**

**Severe, serious and fatal adverse events by subgroups**

|  | Anti-acid medication  at baseline | | No anti-acid medication  at baseline | |
| --- | --- | --- | --- | --- |
|  | Nintedanib (n=244) | Placebo (n=162) | Nintedanib (n=394) | Placebo (n=261) |
| Severe adverse event(s)* | 84 (34.4) | 44 (27.2) | 90 (22.8) | 55 (21.1) |
| Progression of IPF^†^ | 15 (6.1) | 19 (11.7) | 21 (5.3) | 11 (4.2) |
| Pneumonia | 8 (3.3) | 5 (3.1) | 8 (2.0) | 7 (2.7) |
| Diarrhoea | 14 (5.7) | 0 (0.0) | 7 (1.8) | 2 (0.8) |
| Acute respiratory failure | 6 (2.5) | 0 (0.0) | 0 (0.0) | 2 (0.8) |
| Myocardial infarction | 5 (2.0) | 0 (0.0) | 2 (0.5) | 1 (0.4) |
| Serious adverse event(s)^‡^ | 87 (35.7) | 60 (37.0) | 107 (27.2) | 67 (25.7) |
| Progression of IPF^†^ | 17 (7.0) | 23 (14.2) | 25 (6.3) | 16 (6.1) |
| Pneumonia | 11 (4.5) | 7 (4.3) | 12 (3.0) | 9 (3.4) |
| Pulmonary hypertension | 6 (2.5) | 3 (1.9) | 5 (1.3) | 6 (2.3) |
| Chest pain | 4 (1.6) | 4 (2.5) | 2 (0.5) | 0 (0.0) |
| Myocardial infarction | 5 (2.0) | 0 (0.0) | 2 (0.5) | 2 (0.8) |
| Acute respiratory failure | 6 (2.5) | 0 (0.0) | 0 (0.0) | 2 (0.8) |
| Squamous cell carcinoma | 1 (0.4) | 4 (2.5) | 0 (0.0) | 1 (0.4) |
| Fatal adverse event(s)^§^ | 20 (8.2) | 15 (9.3) | 17 (4.3) | 16 (6.1) |
| Progression of IPF^†^ | 8 (3.3) | 9 (5.6) | 10 (2.5) | 7 (2.7) |
| Pneumonia | 4 (1.6) | 1 (0.6) | 1 (0.3) | 1 (0.4) |
| Respiratory failure | 2 (0.8) | 2 (1.2) | 0 (0.0) | 0 (0.0) |
| Respiratory tract infection | 0 (0.0) | 1 (0.6) | 1 (0.3) | 1 (0.4) |
| Myocardial infarction | 2 (0.8) | 0 (0.0) | 0 (0.0) | 1 (0.4) |
| Cardiac arrest | 0 (0.0) | 1 (0.6) | 0 (0.0) | 1 (0.4) |
| Cardiorespiratory arrest | 0 (0.0) | 1 (0.6) | 0 (0.0) | 0 (0.0) |
| Hypoxia | 0 (0.0) | 1 (0.6) | 0 (0.0) | 0 (0.0) |

*Severe adverse events in ≥2% of patients in any of the subgroups shown. A severe adverse event was defined as an event that was incapacitating or that caused an inability to work or to perform usual activities. ^†^Corresponds to MedDRA term ‘IPF’, which included disease worsening and acute exacerbations of IPF. ^‡^Serious adverse events in ≥2% of patients in any of the subgroups shown. A serious adverse event was defined as an event that resulted in death, was immediately life-threatening, resulted in persistent or clinically significant disability or incapacity, required or prolonged hospitalization, was related to a congenital anomaly or birth defect, or was deemed serious for any other reason. ^§^Fatal adverse events in ≥0.5% of patients in any of the subgroups shown.
